# Bioinformatics analysis of the molecular mechanism of obesity in polycystic ovary syndrome

**DOI:** 10.18632/aging.202938

**Published:** 2021-04-27

**Authors:** Jiaojiao Zhou, Xiaolin Huang, Bingshuang Xue, Yuhe Wei, Fei Hua

**Affiliations:** 1 Department of Endocrinology, The Third Affiliated Hospital of Soochow University, Changzhou, Jiangsu 213003, China; 2 Department of Endocrinology, The Affiliated Wujin Hospital of Jiangsu University, Changzhou, Jiangsu 213017, China

**Keywords:** PCOS, obesity, bioinformatics, CHRDL1

## Abstract

Background: Obesity is an important part of polycystic ovary syndrome (PCOS) pathologies. The present study utilized the bioinformatics method to identify the molecular mechanism of obesity status in PCOS.

Methods: Six transcriptome profiles of adipose tissue were obtained from online databases. The background correction and normalization were performed, and the DEGs were detected with the settings *p* < 0.05. The GO, KEGG pathway enrichment, and PPI network analysis were performed with the detected DEGs.

Results: A total of 37 DGEs were found between obesity PCOS and healthy controls, and 8 of them were tested significant in the third database. The expression patterns of the 8 detected DGEs were then measured in another two datasets based on lean/obesity PCOS patients and healthy controls. The gene CHRDL1 was found to be in linear regression with the BMI index in PCOS patients (*p* = 0.0358), but such a difference was not found in healthy controls (*p* = 0.2487). The expression of CHRDL1 was significantly higher in obesity PCOS cases than the BMI matched healthy controls (*p* = 0.0415). Further enrichment research demonstrated the CHRDL1 might function as an inhibitor of the BMP4 or IGF1 signalling.

Conclusion: In summary, the present study identified CHRDL1 as a candidate gene responsible for the obesity of PCOS patients.

## INTRODUCTION

Polycystic ovary syndrome (PCOS), a gender-specific disease, is one of the most common endocrine disorders in women of reproductive age. The disease affects about 4-18% of the total population and is one of the leading causes of female poor fertility. The disease is characterized by multiple cysts of the ovaries but also has multiple symptoms like obesity, infertility, hyperandrogenism, and insulin resistance [1, 2]. The pathologies of PCOS are not well understood, nor is there an effective treatment. Moreover, the causal relationship between reproductive and metabolic features in PCOS has not been fully elucidated yet. It was once believed that the reproductive system was the main reason for developing metabolic disorders, but nowadays, metabolic disorders are widely believed to play an important role in inducing reproductive problems. For example, insulin resistance (IR) is thought to be a key pathophysiological feature contributing to both reproductive and other metabolic disturbances in PCOS [3]. The insulin resistance in PCOS is usually resulted from abnormal insulin signalling, metabolic dysfunction in insulin-responsive tissues, and importantly, the increased volume of fat tissues [4, 5].

Obesity is a common and important part of PCOS pathologies, with about 40% of PCOS patients suffer from overweight [6]. The adipose tissue could secret several mediators like cytokines and adipokines and results in insulin resistance and low-grade chronic inflammatory, then contribute to the pathologies of the ovary in turn [7, 8]. However, not all PCOS patients suffer from obesity, and not all obese women suffer from PCOS. Moreover, the decreased fat volume can effectively protect PCOS patients [9–12]. What makes some PCOS patients get a large volume of fat, but the others are not still far from being understood.

The present study aims to identify the potential genes responsible for the obesity of PCOS patients. We first identified the common differentially expressed genes (DEGs) from multiple microarrays of obese PCOS patients and age & BMI matched controls. Then, the identified genes were confirmed in a third dataset. The expression of the common DGEs was analyzed again in a unique dataset based on lean PCOS patients to figure out the potential genes responsible for the difference between obesity and lean PCOS patients. Then, the linear regression analysis was performed between the genes and BMI index, and finally, the gene CHRDL1 was found potentially responsible for the obesity status of PCOS patients. However, the relationship between CHRDL1 and PCOS or obesity was rarely researched. We then utilized the PPI network to predict the mechanisms of CHRDL1 in inducing obesity in PCOS patients.

## METHODS

### Data source

**Figure 1:**
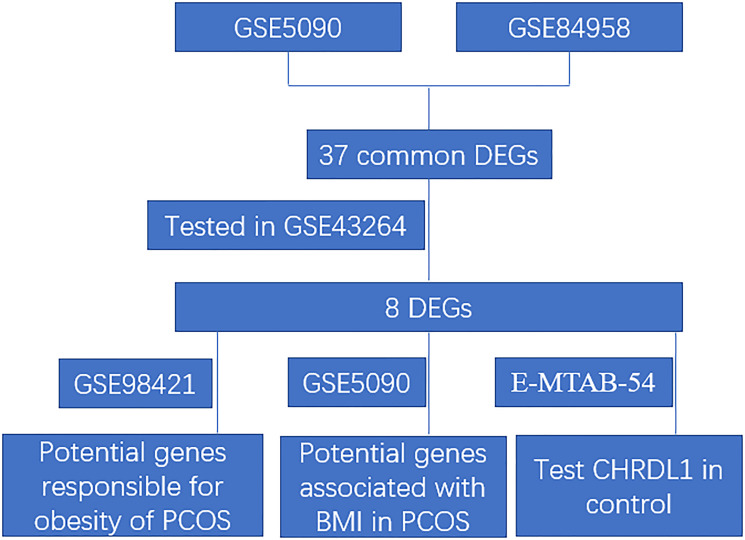
The analytic workflow of the present study.

The original datasets comparing the gene expression profiles of the adipose tissues between PCOS patients and normal controls were downloaded from NCBI GEO databases and ArrayExpress databases (Table 1). The accession number was GSE5090 [13], GSE98421 (https://www.ncbi.nlm.nih.gov/geo/query/acc.cgi?acc=GSE98421), GSE43264 (https://www.ncbi.nlm.nih.gov/geo/query/acc.cgi?acc=GSE43264), GSE84958 [14], E-MTAB-3768 (https://www.ebi.ac.uk/arrayexpress/experiments/E-MTAB-3768/), and E-MTAB-54 (https://www.ebi.ac.uk/arrayexpress/experiments/E-MTAB-54/) respectively. The microarray data of GSE5090 was based on GPL96 ([HG-U133A] Affymetrix Human Genome U133A Array), GSE98421 and E-MTAB-54 were based on GPL570 (Affymetrix Human Genome U133 Plus 2.0 Array), GSE43264 was based on GPL15362 (NuGO array (human) NuGO_Hs1a520180), GSE84958 was based on GPL16791 (Illumina HiSeq 2500), and E-MTAB-3768 was based on A-MEXP-2072 (Illumina HumanHT-12_V4). The clinic information of the E-MTAB-3768 dataset was also downloaded, especially the BMI index. The analytic workflow was shown in Figure 1.

**Table 1:** The basic backgrounds of the datasets included.

	**Platforms**	**Country**	**Year**	**Samples**	**Population**
GSE5090	[HG-U133A] Affymetrix Human Genome U133A Array	Spain	2006	adipose tissue	PCOS and control
GSE98421	Affymetrix Human Genome U133 Plus 2.0 Array	USA	2017	adipose tissue	Lean PCOS females and control
GSE43264	NuGO array (human) NuGO_Hs1a520180	Ireland	2014	adipose tissue	PCOS and control females
GSE84958	Illumina HiSeq 2500	UK	2016	adipose tissue	PCOS and control females
E-MTAB-3768	Illumina HumanHT-12_V4	USA	2014	adipose tissue	PCOS and control females
E-MTAB-54	Affymetrix Human Genome U133 Plus 2.0 Array	UK	2010	adipose tissue	Obese non-PCOS cases

### Data pre-processing and differential expression analysis

The Robust multi-array average (RMA) and FPKM (Fragments Per Kilobase per Million) approach were performed for background correction and normalization of the raw data of the datasets GSE5090 and GSE84958. Then, the differentially expressed genes (DEGs) were detected using the limma R package. The DGEs were defined only with the setting *p* < 0.05 to detect all the potential genes as much as possible based on Benjamini and Hoch-Berg (BH) procedure. Intersect function in R was used for identifying the common DEGs between GSE5090 and GSE84958. The Venn diagram was generated by the online software in Bioinformatics & Evolutionary Genomics (http://bioinformatics.psb.ugent.be/webtools/Venn/). The expression of the detected common genes was then re-analyzed in the third dataset (GSE43264) to confirm the results. The limma R package was used to analyze the difference between the patients and controls, and the results were shown by GraphPad Prism software. The heatmap was also drawn by the online tool Morpheus (https://software.broadinstitute.org/morpheus/).

### Linear regression of candidate genes and BMI index in PCOS

The expression value of the detected genes was extracted from the dataset E-MTAB-3768; then, the linear regression analysis was performed between the expression values of these genes and the BMI index of every sample. The GraphPad Prism software was used to calculate the significant level and regression coefficient.

### KEGG enrichment analysis

Kyoto Encyclopedia of Genes and Genomes (KEGG) enrichment analysis was performed using the online database KOBAS (a web server for gene/protein functional annotation and functional gene set enrichment, http://kobas.cbi.pku.edu.cn/kobas3). The enriched pathways were obtained to analyze the common DEGs at the functional level. *P* < 0.05 was set as the threshold value.

### PPI network construction

GeneMANIA online database (http://genemania.org/) was used to analyze and visualize the protein-protein interaction (PPI) of DEGs and predict the mechanism of CHRDL1 in obesity of PCOS.

## RESULTS

### The DEGs between obesity PCOS cases and age and BMI matched healthy controls

**Figure 2:**
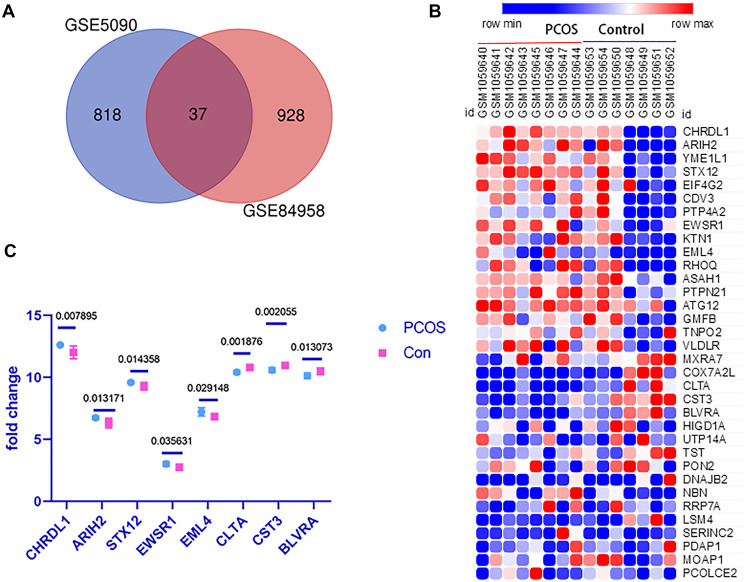
**The DEGs between obesity PCOS cases and age & BMI matched healthy controls.** (**A**) The Venn diagram of common genes between GSE5090 and GSE84958. (**B**) The expression heatmap of the detected DGEs in dataset GSE43264. (**C**) The expression pattern of the 8 significantly differentially expressed genes.

The DGEs of datasets GSE5090 and GSE84958 were analyzed with DECenter. A total of 855 and 965 DGEs were detected from GSE5090 and GSE84958, respectively. A total of 37 common genes were found between GSE5090 and GSE84958, as shown in the Venn diagram of Figure 2A. Then, we tried to confirm the expression pattern of these detected DGEs in another dataset, GSE43264. As shown in the heatmap (Figure 2B), only 8 of them were statistically significant (Figure 2C).

### The DGEs special for obesity in PCOS

**Figure 3:**
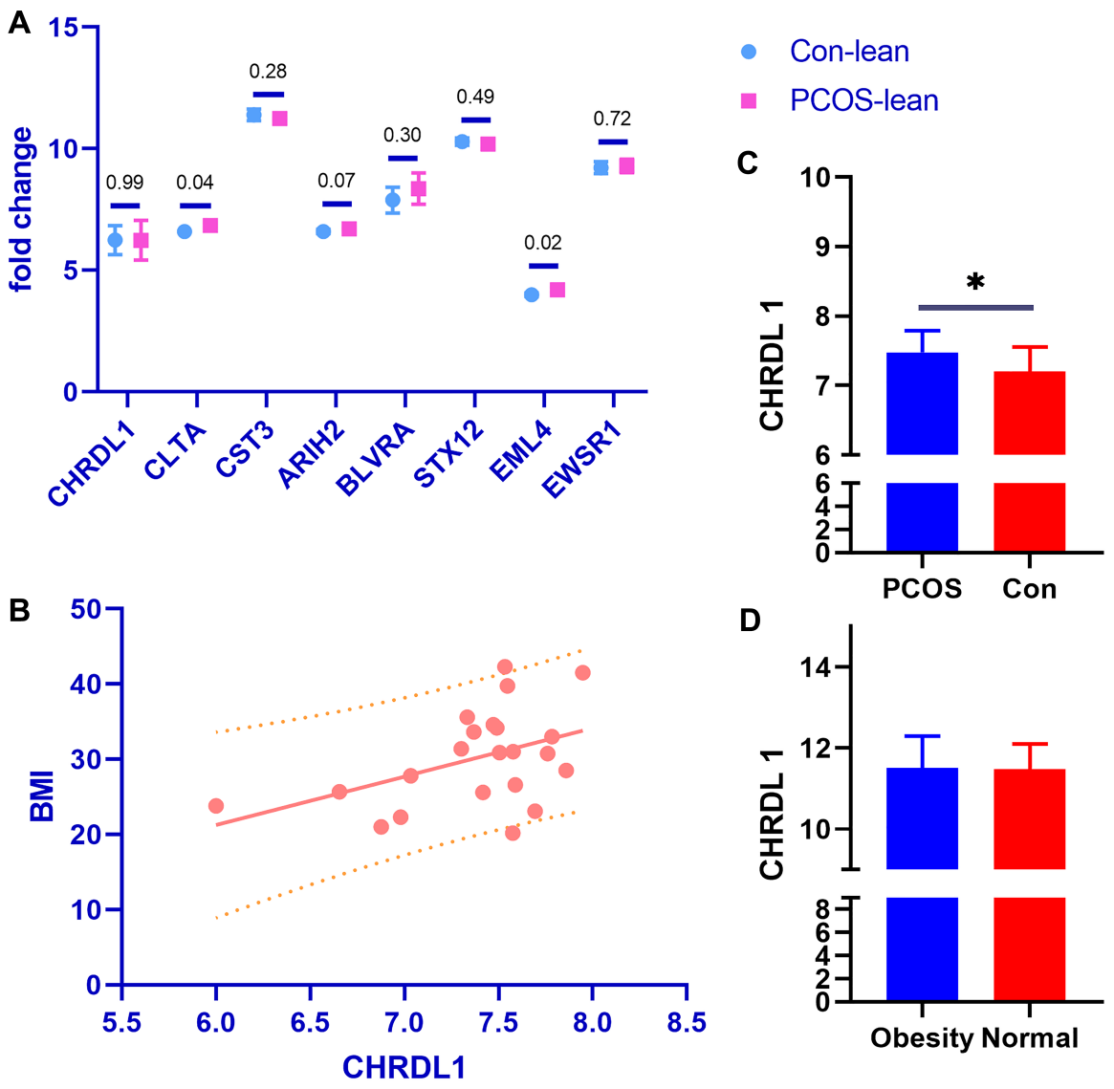
**The DGEs special for obesity in PCOS.** (**A**) The expression pattern of the 8 detected DGEs in GSE98421. (**B**) The expression of the gene CHRDL1 was found to be in linear regression with the BMI index in PCOS patients (*p* = 0.0358, Y = 6.460^*^X - 17.51). (**C**) The expression of CHRDL 1 was significantly higher in obesity PCOS cases than the BMI matched healthy controls (*p* = 0.0415). (**D**) There was no statistical difference of CHRDL1 expression between the obesity and normal cases.

Though obesity is a common characteristic of PCOS, lean patients are not rare in the clinic. We then explored the main DGEs between obese and lean PCOS patients. First, we explored the expression pattern of the 8 detected DGEs in GSE98421, a dataset based on lean PCOS patients and age & BMI matched healthy controls. Interestingly, only two of them were statistically significant (CLTA, EML4), and the remaining not (CHRDL1, CST3, ARIH2, BLVRA, STX12, EWSR1) (Figure 3A). It seems these 6 genes may be related to the weight of PCOS patients; thus, we use the dataset E-MTAB-3768 to identify if the expression of these genes is connected with the BMI index. Among the 6 genes, only the expression of gene CHRDL1 was found to be in linear regression with the BMI index in PCOS patients (*p* = 0.0358, Y = 6.460^*^X - 17.51) (Figure 3B), but such difference was not found in the healthy controls (*p* = 0.2487). Similarly, the express ion of CHRDL 1 was significantly higher in obesity PCOS cases than the BMI matched healthy controls (*p* = 0.0415, Figure 3C). Moreover, when only considering the obesity status but regardless of the PCOS, there was no statistical difference of CHRDL 1 expression between the obesity and normal cases, as based on the analysis of the dataset E-MTAB-54 (*p* = 0.8816, Figure 3D).

### Associations between genome-wide expression profiles and CHRDL1 expression

**Figure 4:**
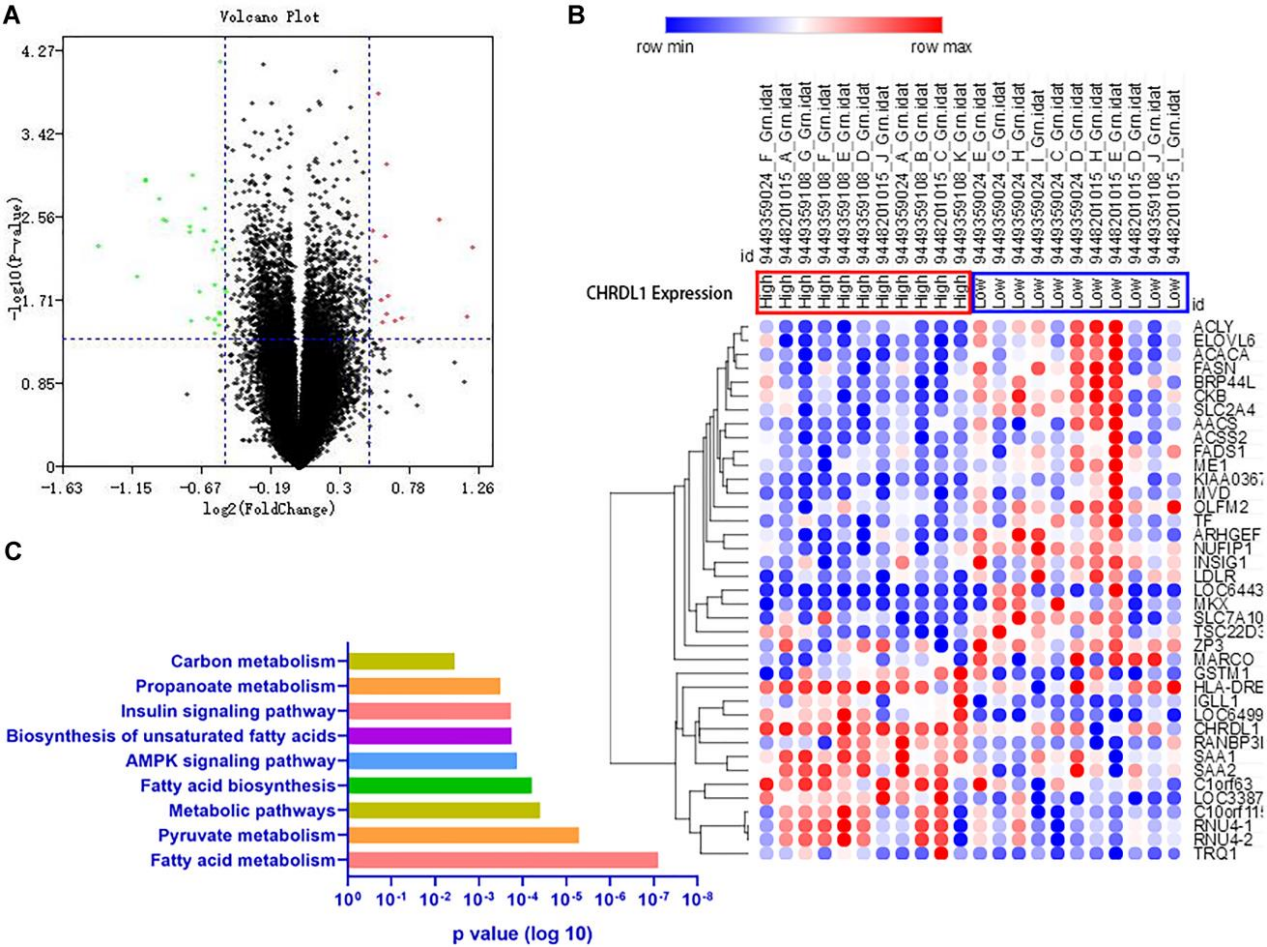
**Associations between genome-wide expression profiles and CHRDL1 expression.** (**A**) The Volcano plots of the gene expression when divided the PCOS cases into two groups according to the CHRDL1 expression. (**B**) The heatmap of the DEGs. (**C**) The KEGG Pathway enrichment analysis of the DEGs.

To further investigate the biological role of CHRDL1 in PCOS, gene expression profiles associated with CHRDL1 were derived based on genome-wide microarray analysis of E-MTAB-3768. The PCOS patients were divided into 2 groups according to the expression level of CHRDL1, and 29 up-regulated and 16 down-regulated genes were identified as significantly associated with CHRDL1 expression between the groups (FDR-adjusted *P* < 0.05 and |logFC| > 0.5, Figure 4A). Further, these aberrant genes were presented as an expression heatmap (Figure 4B). These dysregulated genes were mainly involved in metabolism pathways as shown by KEGG analysis, including fatty acid metabolism, pyruvate metabolism, metabolic pathways, fatty acid biosynthesis, AMPK signalling pathway, biosynthesis of unsaturated fatty acids, insulin signaling pathway, propanoate metabolism, and others, (Figure 4C), indicating the potential role of CHRDL1 in regulating metabolism status in PCOS patients.

### The potential function of CHRDL1 predicted by the PPI network

**Figure 5:**
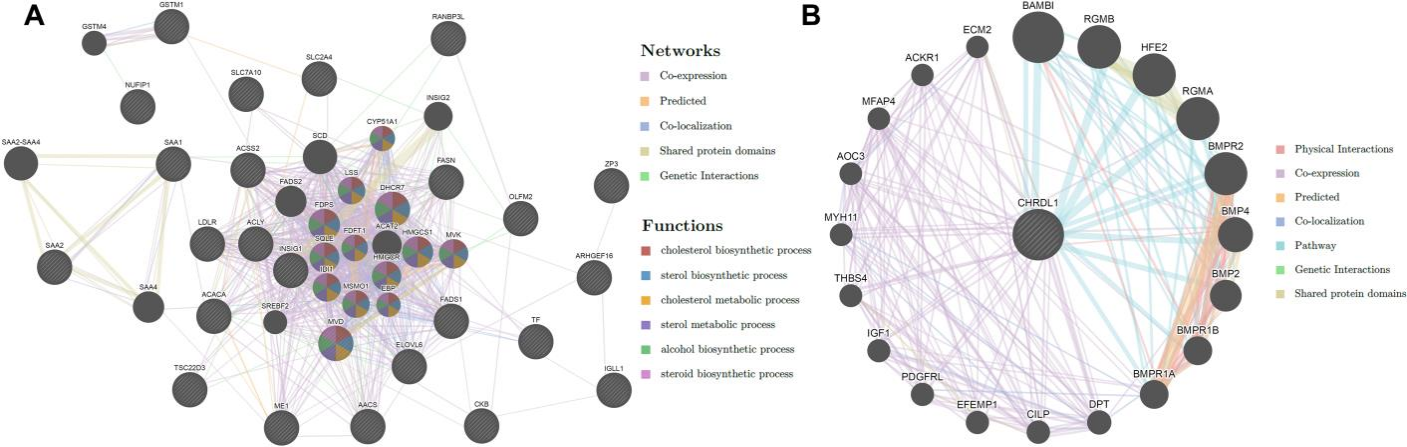
**The potential function of CHRDL1 is predicted by the PPI network.** (**A**) The PPI network of DEGs. (**B**) The proteins associated with CHRDL1 by physical interaction, co-expression, predicted, co-localization, pathway, genetic interactions, and shared protein domains methods.

The gene CHRDL1 is found broadly expressed in fat, prostate and 15 other tissues, acts as an antagonist of bone morphogenetic protein 4 (BMP4). However, the role of CHRDL1 in PCOS and metabolism is rarely investigated. We utilized the protein-protein-interaction (PPI) network by GeneMANIA to analyze the DEGs, and predict the mechanism of CHRDL1 in inducing obesity in PCOS patients. The PPI network of DEGs was constructed and shown in Figure 5A. Similarly, the most significant enriched functions were focused on metabolic processes like cholesterol biosynthetic, sterol biosynthetic, and others, Figure 5B demonstrated the proteins associated with CHRDL1 by physical interaction, co-expression, predicted, co-localization, pathway, genetic interactions, and shared protein domains methods. It is worth noting that the proteins detected were mainly concentrated on BMP signalling, but the relationship between BMP and PCOS was also rarely researched. Another protein that should be noticed was IGF-1, an important part of the pathologies of PCOS.

## DISCUSSION

The present study aimed to identify the potential genes responsible for the obesity status in PCOS patients. We first identified the common DEGs from multiple microarrays of obese PCOS patients and age & BMI matched controls; then, the identified genes were confirmed in a third dataset. The expression of the common DGEs was analyzed again in a unique dataset based on lean PCOS patients to figure out the potential genes responsible for the difference between obesity and lean PCOS patients. Then, the linear regression analysis was performed between the genes and BMI index, and finally, the gene CHRDL1 was found potentially responsible for the obesity status of PCOS patients, which was not for non-PCOS cases. However, the relationship between CHRDL1 and PCOS/obesity was rarely researched. We then utilized the PPI network to predict the mechanism of CHRDL1 in inducing obesity of PCOS patients, and the BMP4 signaling and IGF1 were identified.

The CHRDL1 gene encodes an antagonist of BMP4 by binding to it and preventing its interaction with receptors. The encoded protein plays a role in topographic retinotectal projection and retinal angiogenesis regulation in response to hypoxia. The CHRDL1 protein is reported to contribute to neuronal differentiation of neural stem cells by altering the fate of neural stem cells from gliogenesis to neurogenesis. It may also play an important role in regulating retinal angiogenesis and skeletogenesis by modulating BMP4 actions. The role of CHRDL1 in PCOS is rarely researched, but it has been related to obesity in a few researches. Smith and et al. demonstrated that CHRDL1 protein could inhibit both white and beige adipogenic commitment and differentiation [15]. CHRDL1 increased during preadipocyte differentiation and was a positive marker of PPARγ induction and adipogenesis. Interestingly, CHRDL1 has also been found in the human adipose tissue secretome, indicating that it may be a circulating protein, but nothing is known about its potential endocrine effects [16].

Considering the close association between CHRDL1 and BMP4, the role of BMP4 in PCOS and obesity may point out the potential role and mechanism of CHRDL1 in PCOS or obesity. BMPs are expressed in a cell-specific manner in the ovary and display spatial and temporal changes in expression depending on the stage of follicular development [17]. BMPs are also able to regulate FSH responsiveness and FSH-induced steroidogenesis, hence contribute to the pathogenesis of PCOS. BMP4 is reported to be mainly produced by thecal interstitial cells [17–20], and the expression of BMP4 mRNA has also been reported in mouse, human, and bovine ovaries granulosa cells [21]. However, another study shown the granulosa cells in PCOS down-regulated the expression of BMP4 [22]. The role of BMP4 in PCOS has not been well described. The BMP4 protein has been reported to inhibit progesterone synthesis and secretion in ovarian and granulosa cells but does not affect estradiol [23]. The damage effect of androgen on ovarian function in experimental PCOS may be mediated by BMP4 signalling [24]. No research has been conducted to investigate the role of BMP4 in adipogenesis in PCOS. However, some researches demonstrated the BMP4 protein might be involved in obesity.

The BMP family was widely known to stimulate fat tissue alteration. In a study by Son et al., serum BMP4 levels were measured in male and female subjects and shown to be associated with adiposity, insulin resistance, and metabolic syndrome [25]. Studies also showed that exogenous administration of BMP4 caused the transformation of fat tissue into a more active variant [26]. Another study showed in the case of obesity, BMP4 was able to increase the transformation of adipose tissue [27]. BMP4 is secreted by mature adipocytes and increases in hypertrophic obesity, thereby eliciting a positive feedback signal to recruit new adipocytes [15]. More interestingly, a recent human study showed that administration of BMP4 inhibitors could result in an improvement in obesity-related diseases and changes in body fat distribution [28]. The mechanism of how BMP4 participates in promoting obesity is not well understood; the role in adipogenesis may be an important reason.

Hypertrophic adipocytes have increased endogenous BMP4 gene expression and secretion. One of the early and central events in white adipocyte commitment is BMP4 signalling, which results in the activation of peroxisome proliferator activated receptor gamma (PPARg) and commitment of preadipocytes [29]. Another study also showed that primary human preadipocytes, isolated from the subcutaneous white adipose tissue, underwent improved differentiation *in vitro* when treated with BMP4 [30, 31]. Moreover, BMP4 also has a role in enhancing beige adipogenesis in human preadipocytes [15], transgenic mice overexpressing bmp4 in white adipose tissue [32], as well as in lean mature mice following BMP4 gene therapy [33]. In summary, the BMP4 protein has been linked with both obesity and PCOS, which may be responsible for the obesity status of PCOS cases. Moreover, considering the findings of this paper, the BMP4 inhibitor CHRDL1 is highly expressed in obesity but not in lean PCOS cases; the CHRDL1 gene may functions differently in normal or PCOS cases. Further studies should be conducted to investigate the actual relationship and mechanism between CHRDL1 and the obesity status of PCOS.

The present study also demonstrated the close relationship between IGF1 and CHRDL1, another important protein in PCOS. But no research about it has been reported till now. The role of IGF1 in PCOS has been widely researched. A significantly higher IGF1 level was found in women with PCOS compared to healthy women [34]. The IGF-I protein in PCOS is able to increase the synthesis of sex hormone-binding globulin and high-density lipoprotein. IGF-1R has the ability to stimulate androgen production by ovarian cells [35]. Evidence suggests that ovarian hyperandrogenism is a result of insulin action on the ovaries, and because of that, it is mediated by IGF-1R [35]. The increased level of IGF-I after the reduction diet had a cardioprotective effect [36]. Further studies should be made to figure out the relationship between CHRDL1 and obesity.

## CONCLUSION

In summary, the present study identified the gene CHRDL1 that may be responsible for the obesity of PCOS patients, but not for non-PCOS cases. It was also suggested that CHRDL1 might function as an inhibitor of the BMP4 signalling or via regulation of IGF1. However, several limitations exist with this study, most importantly, the present study was based on bioinformatic analysis, but not functional; further experimental and functional studies need to be performed for exploring the role of CHRDL1 in obesity of PCOS. Moreover, the study was based on several datasets based on different populations and different platforms; it was not possible to combine different datasets to perform an integrated analysis.
